# Supplementary material for: The Roles of AGTRAP, ALKBH3, DIVERSIN, NEDD8 and RRM1 in Glioblastoma Pathophysiology and Prognosis
Source: Biomedicines. 2024 Apr 22;12(4):926. doi: 10.3390/biomedicines12040926 (PMC11048029; doi:10.3390/biomedicines12040926)
Supplement: Supplementary file 1 [file biomedicines-12-00926-s001.zip › biomedicines-2949412-supplementary.pdf]

NEDD8n expression

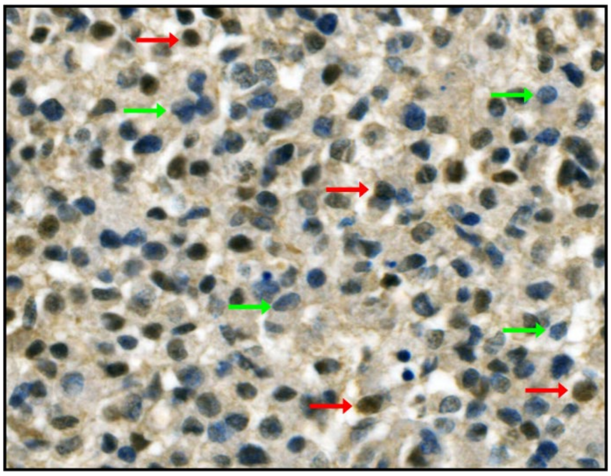

Supplementary Figure S1. NEDD8n expression in GBM tissues. High magnification micrograph showing NEDD8n positive (red arrows) and negative (green arrows) tumor cells.

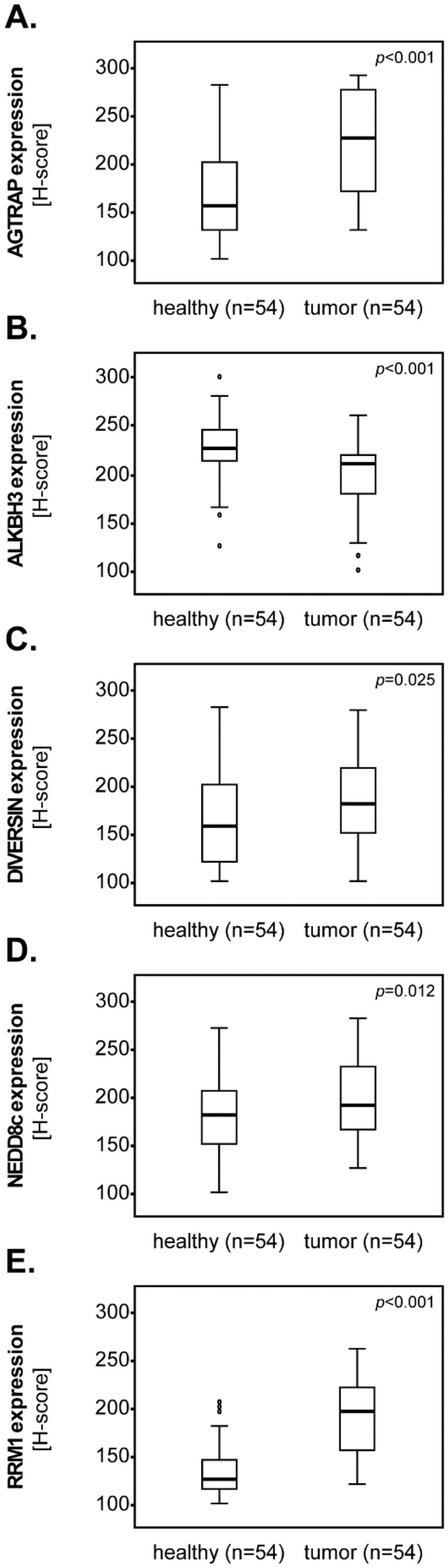

Supplementary Figure S2. Marker expression in healthy versus GBM tissues (n=54). Expression of **(A)** AGTRAP, **(B)** ALKBH3, **(C)** DIVERSIN, **(D)** NEDD8c and **(E)** RRM1 only in the 54 patients where both tumor and non-malign brain tissues were available. The medians are shown as black lines and the percentiles (25th and 75th) as vertical boxes with error bars. Statistical analysis was performed with the Mann-Whitney U test and the *p*-values are indicated in the upper-right corner of each plot.
